# Hygrometrical Tables, to Be Used with, and Description of the Dry- and Wet-Bulb Thermometers

**Published:** 1847-07

**Authors:** 


ART. V.—*Hygrometrical Tables, to be used with, and Description of the Dry- and Wet-Bulb Thermometers.* By JAMES GLAISHER, Esq., of the Royal Observatory, Greenwich.—*London,* 1847. 8vo, pp. 45.

THOSE who are interested in medical topography are well aware how scanty our knowledge is of the humidity of the air. The quantity of rain which falls, and the number of rainy days, are nearly all the data we have at present of the humidity of most climates; and yet the influence of this condition on the human body is confessedly great, and any exact information promises much benefit. On this account we call attention to this pamphlet, which describes a more simple method of ascertaining the principal facts connected with the moisture of the atmosphere than has yet been adopted. The hygrometers made of catgut, wood, hair, whalebone, &c. are easily deranged, and cannot be relied on. Daniel's hygrometer requires the purest kind of ether, and much experience to use it effectively. The instrument now proposed is merely a common thermometer, the bulb of which is covered with muslin and kept wet with cold water by a simple contrivance. A second thermometer, with a dry bulb is necessary, as the humidity of the air is ascertained by comparing the temperature of the one with the other. If the air is saturated with moisture, no evaporation takes place, and there is no difference between the two thermometers; but if the air is dry evaporation takes place, cold is produced, the wet-bulb thermometer sinks, and its fall is in proportion to the dryness of the air. Having ascertained the two temperatures, that of the dry, and that of the wet-bulb, the observer has only to consult the tables calculated by Mr. Glaisher, and he at once ascertains various particulars regarding the humidity of the air.

For instance:—If the temperature be alike in both, then the air is saturated with moisture; but if the dry-bulb is 70° and the wet-bulb 55°, then the table shows that the degree of humidity is 0·470, that of complete saturation being represented by unity. Another column shows that under these circumstances the weight of the vapour in a cubic foot of air is 3·76 grains, and as it takes 8 grains of water to saturate a cubic foot of air, there will be required 4·24 grains of water more, to complete saturation. Another column gives the dew-point, and a fourth the height of a column of mercury, which the vapour will support.

The application of this instrument to the sick room is obvious. If the air is very dry, the difference between the two instruments will be great; if moister, there will be less difference, and when the air is saturated with moisture there will be no difference at all. If the difference between the two is very great, it may be necessary to make a moister atmosphere by exposing water in shallow vessels; or if a more rapid effect is required, by using heated water until the air is sufficiently moist, and then by replacing it with cold. If the air is too moist, or a dry air is needed, all water should be removed or covered, and some substance, like sulphuric acid which rapidly absorbs water, should be placed in the room until the requisite dryness is obtained.

Mr. Glaisher alludes to the value of information which may be obtained by this instrument, as to the humidity of the air in various places and times, in connexion with the mortality, and in addition to the Reports on Mortality of the Registrar-General. On this account, as well as for its use in the sick-room, we give this slight notice, referring those who are likely to take the necessary pains in registering the particulars of the climate in which they reside, to Mr. Glaisher's pamphlet, which, besides the tables, contains very full directions for the employment of the thermometer, both in a wet and dry state. The papers on Medical Topography, in the Transactions of the Provincial Medical Association, indicate that there are many careful observers scattered over the kingdom, who would rejoice in any practical hints as to the best mode of conducting their inquiries. We would suggest, that such careful daily records might be best kept by many of those who are retired from the more active duties of their profession (either nolentes or volentes), many of whom might find in such constantly and regularly recurring observations requiring accuracy and time, a stated employment, whilst the reductions of these, so as to obtain some useful results, would satisfy and keep in practice the higher powers of their minds.

